# Stoichiometric and nutrient resorption characteristics of dominant tree species in subtropical Chinese forests

**DOI:** 10.1002/ece3.3527

**Published:** 2017-11-15

**Authors:** Yelin Zeng, Xi Fang, Wenhua Xiang, Xiangwen Deng, Changhui Peng

**Affiliations:** ^1^ Faculty of Life Science and Technology Central South University of Forestry and Technology Changsha China; ^2^ Huitong National Field Station for Scientific Observation and Research of Chinese Fir Plantation Ecosystem in Hunan Province Huitong China; ^3^ Department of Biological Sciences Institute of Environment Sciences University of Quebec at Montreal Montreal QC Canada

**Keywords:** nutrient use strategy, resorption efficiency, resorption proficiency, stoichiometry ratios, successional stage

## Abstract

This study investigated seasonal patterns in stoichiometric ratios, nutrient resorption characteristics, and nutrient use strategies of dominant tree species at three successional stages in subtropical China, which have not been fully understood. Fresh leaf and leaf litterfall samples were collected in growing and nongrowing seasons for determining the concentrations of carbon (C), nitrogen (N), and phosphorus (P). Then, stoichiometric ratios (i.e., C:N, C:P, N:P, and C:N:P) and resorption parameters were calculated. Our results found that there was no consistent variation in leaf C:N and C:P ratios among different species. However, leaf N:P ratios in late‐successional species became significantly higher, indicating that P limitation increases during successional development. Due to the P limitation in this study area, P resorption efficiency and proficiency were higher than corresponding N resorption parameters. Dominant tree species at early‐successional stage adopted “conservative consumption” nutrient use strategy, whereas the species at late‐successional stage inclined to adopt “resource spending” strategy.

## 1 INTRODUCTION

Concentrations of carbon (C), nitrogen (N), and phosphorus (P) in foliage and their variation according to tree species, plant phenology, and soil environment are important for plant growth and nutrient cycling in forest ecosystems (Eckstein & Karlsson, 1997; Hagen‐Thorn, Armolaitis, Callesen, & Stjernquist, 2004; Han, Fang, Guo, & Zhang, 2005; He et al., 2008; McGroddy, Daufresne, & Hedin, 2004). C, N, and P are essential nutrient elements that support plant growth and are closely correlated with several significant metabolic processes, including carbohydrate synthesis, photosynthesis, respiration, and lipometabolism (Pan, 2006). Nutrient concentrations in foliage, their seasonal variations, and the tendency of variation from fresh leaves to litterfall result from the adaptation of plants to environmental stresses and in turn influence soil nutrient concentrations in forests (Hagen‐Thorn et al., 2004; Salehi, Ghorbanzadeh, & Salehi, 2014; Victor, Dimitrios, Alexandros, Georgios, & Georgios, 2001; Wardle, Walker, & Bardgett, 2004).

The ecological stoichiometry of the elements C, N, and P is a prime area for the investigation of elemental balance in ecosystems, which provides an integrative nutrient framework linking biogeochemical patterns on a global scale to physiological constraints at the cellular levels (Clevelan & Liptzin, 2007; Elser et al., 2000; McGroddy et al., 2004). Ecological stoichiometry is considered more valuable than individual nutrient concentrations for understanding the stability of an entire ecosystem (Sterner & Elser, 2002). The N:P ratio in fresh leaves has been widely accepted as a reliable indicator of nutrient limitations. N:P ratios less than 14 and N concentrations lower than 20 mg/g reflect N limitation; N:P ratios greater than 16 and P concentrations lower than 1.0 mg/g reflect P limitation; and N:P ratios between 14 and 16 reflect N and P colimitation (Koerselman & Meuleman, 1996; Tessier & Raynal, 2003; Verhoeven, Koerselman, & Meuleman, 1996). N:P ratios are not only related to the nutrient concentrations in soil, but are also influenced by climatic zone (McGroddy et al., 2004; Reich & Oleksyn, 2004), tree species, plant life forms (Aerts & Chapin, 2000; Killingbeck, Hammenwinn, Vecchio, & Goguen, 2002), and successional gradients (Yan, Wang, & Huang, 2006). Zhang's experiments in Karst regions (Zhang et al., 2015) and several other studies (Davidson et al., 2007; Du, Pan, Li, Hu, & Wang, 2011; Huang et al., 2013) have indicated that foliar N:P ratios may change with succession owing to the N cycling properties recover accompanying with P limitation increases in mature forests. The stoichiometric ratios may also vary seasonally, because nutrient concentrations in leaves change with phenology, particularly in deciduous species (Regina, Rico, Rapp, & Gallego, 1997; Robert, Caritat, Bertoni, Vilar, & Molinas, 1996). However, published literatures focus more on stoichiometric change patterns on a large scale, such as in the world's temperate forests or tropical rain forests (Han et al., 2005; He et al., 2006, 2008; McGroddy et al., 2004; Reich & Oleksyn, 2004), whereas research on stoichiometry variations with seasons and successions among different tree species in individual forest or on a small scale is less developed.

Nutrient resorption, defined as nutrient translocation from senescing tissues and reuse in newly grown tissues, greatly affects nutrient cycling at the ecosystem scale and has significant implications for nutrient use strategy, plant growth, and competitive ability at the individual plant level (Killingbeck, 1996; Pugnaire & Chapin, 1993; Wang & Moore, 2014). Resorption is almost as important as nutrient uptake from the environment, which reduces dependence on soil nutrient availability and on the uptake of nutrients by roots (Aerts, 1996; Côté, Fyles & Djalilvand, 2002). Resorption efficiency (RE), defined as the resorption of the nutrient retranslocated to live tissues before senescence and reflecting plant physiology and metabolic processes, is commonly used to measure nutrient resorption (Chapin, 1980). Another commonly used parameter is resorption proficiency (RP), which is defined as the level of nutrients reduced in leaf litterfall and is directly linked with the decomposition process (Killingbeck, 1996). N concentrations in litterfall lower than 7.0 mg/g or P concentrations lower than 0.5 mg/g were considered to have a high RP of N or P, respectively. Overall, studies related to plant nutrient resorption have largely focused on its relationship to soil nutrient availability (Milla, Palacio‐Blasco, Maestro‐Martinez, & Montserrat‐Marti, 2006; Yuan & Wan, 2005), plant life forms (Aerts, 1996; Yuan, Li, Han, Huang, & Jiang, 2005), the succession of forests (Kazakou, Garnier, Roumet, Collin, & Laurent, 2007; Yan et al., 2006), and leaf lifespan (Eckstein, Karlsson, & Weih, 1999; Wright & Cannon, 2001); most have compared resorption performance in terms of plant adaptations to nutrient‐poor fields and nutrient‐rich fields (Kobe, Lepczyk, & Iyer, 2005). Although popular opinion holds that plants in nutrient‐poor environments are more likely to have higher resorption abilities than plants from nutrient‐rich environments, existing results on the relationship between plant resorption and soil nutrient availability are inconsistent (Aerts, 1996; Aerts & Chapin, 2000; Norris & Reich, 2009). Moreover, studies of nutrient resorption characteristics among tree species in similar climatic and environmental conditions have been limited.

The nutrient use strategy of a species may reflect the coevolution of foliage nutrient concentrations and soil environments (Killingbeck, 1996), and nutrient resorption is the most important element of this process. Several previous studies have shown that tree species from infertile fields usually have low leaf nutrient concentrations, high nutrient resorption values, and slow litter decomposition rates; that is, they adopt a “conservative consumption” nutrient use strategy (Aerts & Chapin, 2000; Escudero, Arco, Sanz, & Ayala, 1992; Kobe et al., 2005; Wright & Cannon, 2001). Tree species from nutrient‐rich fields usually have high nutrient concentrations in fresh leaves and low nutrient REs from litterfall; that is, they adopt a “resource spending” nutrient use strategy (Aerts & Chapin, 2000; Reich, Walters, & Ellsworth, 1992; Wright & Cannon, 2001). As to the situation of ecological succession, the classic concept of nutrient strategies established by Odum (1969) hypothesized that the biogeochemical cycling of major nutrients tends to closing or tightening. That is, compared to developing systems, the mature ones have a greater capacity to entrap and hold nutrients for cycling within the system. This hypothesis was supported by some empirical studies, in which the results showed that the early‐successional species tend to have lower resource uptake and loss rate (Garnier et al., 2004; Vile, Shipley, & Garnier, 2006). However, results from a study of evergreen broad‐leaved forests in Tiantong National Forest Park, eastern China, showed the opposite tendency to Odum's hypothesis; that is, the leaf and soil nutrient concentrations increased along the succession gradient, and the nutrient use strategies of dominant tree species shifted from “conservative consumption” through “intermediate” to “nutrient spending.” That report also noted that nutrient use strategy may differ depending on the different functional types of tree species, so it is still unclear whether the same results would apply to other regions (Yan et al., 2006).

Evergreen broadleaved forests are climax vegetation in the subtropical area of southern China, which has more favorable climatic conditions than other regions of the same latitude (Zeng et al., 2014). Over the last decades, original evergreen broadleaved forests have largely converted into secondary forests or replaced with plantations. Secondary forest restoration reflects a natural succession process in which the tree species composition changes from conifers through deciduous broadleaved species to evergreen broadleaved species. Therefore, this region contains a diversity of tree species and has been seen as a template for the sustainable management of mixed plantations and natural forests. It provides an ideal research field for investigating the stoichiometric and nutrient resorption characteristics of many different tree species under similar environmental conditions.

In this study, five dominant tree species (*Cunninghamia lanceolata*,* Pinus massoniana*,* Choerospondias axillaris*,* Cyclobalanopsis glauca,* and *Lithocarpus glaber*), representing plantation and three successional secondary forests were selected to investigate the variations of nutrient stoichiometric ratios in leaves change with seasons and successional stages. Then, the nutrient resorption parameters (RE and RP) were calculated to compare the resorption characteristics and nutrient use strategies among tree species at different successional stages. Specifically, we tested the following hypotheses:

1. The variation pattern of leaf stoichiometry would be C:N ratios decrease, while C:P and N:P ratios increase from early‐ to late‐successional species.
2. Nutrient resorption characteristics of tree species would be highly associated with nutrient limitation.
3. Considering dominant tree species, nutrient use strategy would shift from “conservative consumption” in early‐successional stage to “resource spending” in late‐successional stage in this study area.

## 2 MATERIALS AND METHODS

### 2.1 Site description

**Figure 1:**
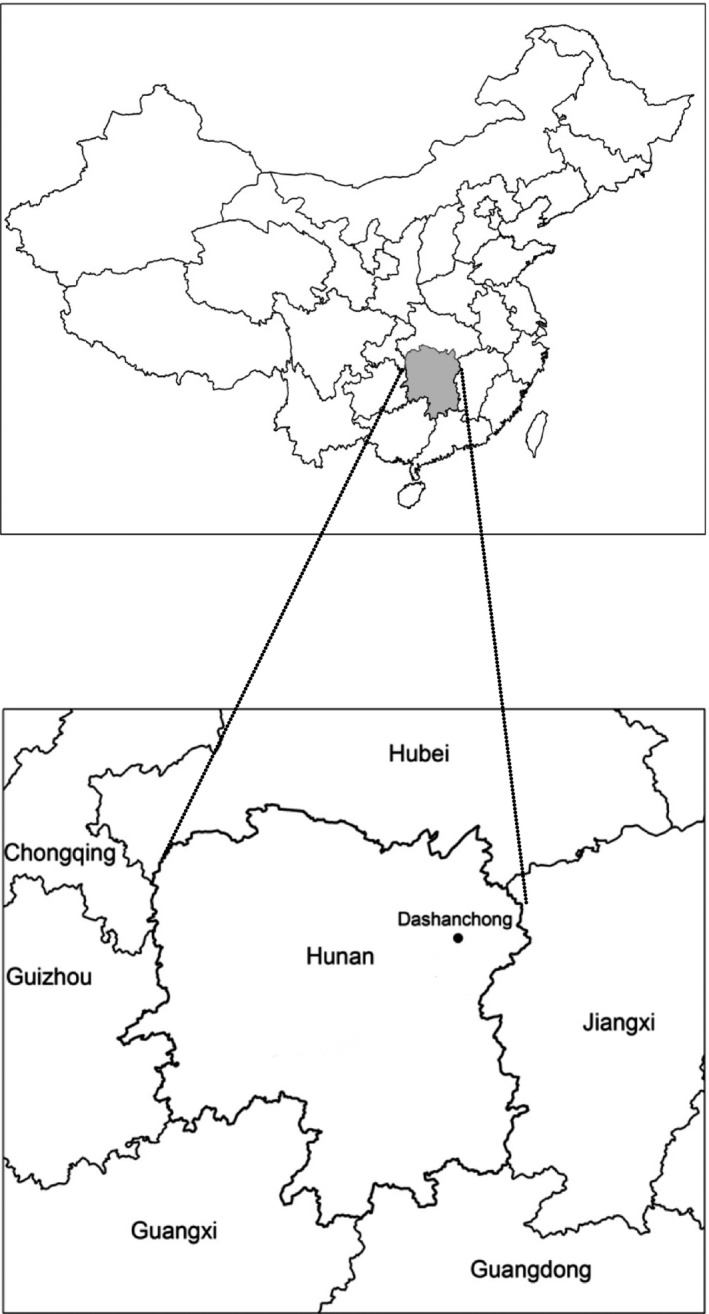
Location of Dashanchong Forest Park in Changsha, Hunan Province, southern China

This study was conducted at Dashanchong Forest Park (28°24′N, 113°18′E) in Changsha County, Hunan Province, China (Figure 1). This area has a typical humid midsubtropical monsoon climate, and the mean annual air temperature is 16.5°C. Average annual precipitation is 1,420 mm, occurring primarily between April and August. The topography is characterized by hilly terrain, with altitudes ranging from 55 to 217.4 m above sea level. The soil type is well‐drained clay loam red soil developed from slate parent rock classified as Alliti‐Udic Ferrosols, corresponding to Acrisol in the World Reference Base for Soil Resources (China Soil Database, http://vdb3.soil.csdb.cn/).

Four forest types in Dashanchong Forest Park were chosen for this study, including one conifer (*Cunninghamia lanceolata*, CL) plantation and three naturally restored forests dominated by coniferous (*Pinus massoniana*, PM), deciduous broadleaved (*Choerospondias axillaris*, CA), and evergreen broadleaved tree species (*Cyclobalanopsis glauca*, CG and *Lithocarpus glaber*, LG). The four forests have a closed canopy structure and a thick intact litter layer. In 2009, a 1‐ha permanent plot was established in each of the four forests. Then, the plots were divided into 10 m × 10 m subplots to map the locations of individual trees and to record tree species, diameter at breast height (1.3 m), height, and crown width. Dominant tree species and nutrient concentrations in surface soils (0–30 cm) in the four forests investigated are provided in Table 1.

**Table 1:** Dominant tree species and soil nutrient concentrations at depths of 0–30 cm in the four forests investigated

Forest type	Dominant species	Importance value (%)	Average tree height (m)	Soil depth (cm)	Organic C (g/kg)	Total N (g/kg)	Total P (g/kg)
CL	*C. lanceolata*	100.00	19.5	0–15	19.72 ± 4.27^b,c^	1.12 ± 0.23^c,d,e^	0.21 ± 0.06^b^
				15–30	14.99 ± 3.36^c^	0.96 ± 0.22^e^	0.20 ± 0.07^b^
PM	*P. massoniana*	28.24	13.5	0–15	24.21 ± 7.49^a,b^	1.37 ± 0.29^b,c^	0.25 ± 0.06^a,b^
	*L. glaber*	20.04	8.0	15–30	17.75 ± 4.19^c^	1.02 ± 0.22^e^	0.22 ± 0.05^b,c^
CA	*C. axillaris*	26.49	15.9	0–15	23.63 ± 6.97^a,b^	1.65 ± 0.44^a^	0.29 ± 0.07^a^
	*Loropetalum chinensis*	15.51	4.8	15–30	18.40 ± 4.62^c^	1.33 ± 0.44^c,d^	0.27 ± 0.06^a^
CG	*C. glauca*	9.90	11.4	0–15	25.79 ± 7.34^a^	1.44 ± 0.36^a,b^	0.20 ± 0.04^c^
	*L. glaber*	25.93	10.5	15–30	18.48 ± 6.71^c^	1.12 ± 0.37^c,d,e^	0.19 ± 0.04^c^

CL, *Cunninghamia lanceolata* forest; PM, *Pinus massoniana* forest; CA, *Choerospondias axillaris* forest; CG, *Cyclobalanopsis glauca;* LG, *Lithocarpus glaber* forest.

In each column, values from the same sample type with different letters indicate significant differences at *p* < .05.

### 2.2 Samples collection and analysis

Soil and leaf samples were collected from July 2012 to June 2013 (1 year) in the four forests. Soil samples were collected separately at depths of 0–15 and 15–30 cm in the central six subplots parallel to the slope within each plot of the forests in March, June, September, and December. In each tree species, leaf samples were collected repeatedly from randomly chosen nine trees of average height (Table 1) parallel to the plot slope, with three in the upper, middle, and lower slope, respectively. Leaf litterfall samples were collected every month using round litter traps each with an area of 1 m^2^. Litterfall samples were retrieved from the litter traps and transported to the laboratory to be separated into several parts, such as leaves of dominant species, other leaves, fine wood, reproductive parts, and miscellaneous parts. The calculation of gross weight of leaf litterfall and the analysis of nutrients concentration were only based on a fraction of dominant species leaves. Fresh leaf samples were collected every month using a tree trimmer in the middle and upper crown from all directions (east, south, west, and north, about 20 g for each direction) in each chosen tree, and then, the leaves from one tree were snipped and mixed to obtain one replicate. All samples were dried at 80°C to a constant weight and ground. Then, all samples were stored in plastic bags and kept at 4°C until chemical analysis was performed. Based on seasonal climate changes in the study area, particularly the phenological phenomenon of the deciduous species CA, we divided the year into two periods to investigate seasonal variation in stoichiometric and nutrient resorption characteristics: the growing season from April to September and the nongrowing season from October to March. Data on fresh leaves of CA in the nongrowing season were omitted, because in this season there were no fresh leaves from deciduous species.

The concentrations of C, N, and P were determined for soil and leaf samples of dominant species (Xiang, Chai, Tian, & Peng, 2009). C concentrations were measured by the wet combustion method using oxidization of potassium bichromate (the Walkley‐Black procedure; Institute of Soil Science, Chinese Academy of Science 1978). N concentrations were measured using the Semimicro‐Kjeldahl method, digested with a mixture of H_2_SO_4_, K_2_SO_4_, CuSO_4_, and Se. P concentrations were determined using the acid‐extracted molybdenum colorimetric method with HCl‐NH_4_F digestion.

### 2.3 Data analysis

Nutrient concentrations in soil, leaf litterfall, and fresh leaf samples were expressed on the basis of the dry weights of soil and leaf litterfall and fresh leaves of dominant species, respectively. Nutrient RE was calculated using the following formula:

$$\text{RE}=\left(1-\frac{{\left[\text{Nutrient}\right]}_{\mathrm{litterfall}}}{{\left[\text{Nutrient}\right]}_{\mathrm{fresh}\mathrm{leaf}}}\right)\times 100\%$$

where [Nutrient]_litterfall_ and [Nutrient]_fresh leaf_ are nutrient concentrations in litterfall samples and fresh leaves, respectively. N and P REs were calculated. The return amounts of N and P were calculated as the gross weight of leaf litterfall multiplied by the concentrations of N and P in litterfall, respectively.

One‐way analysis of variance was carried out to determine the effects of tree species on all variables. The least significant difference (LSD, *p* < .05) test was used to separate treatment means. Significant differences between mean stoichiometry values of leaf litterfall and fresh leaves in the growing and nongrowing seasons were tested using Student's *t* test (at a significance level of 0.05). The correlations among N and P REs, N:P stoichiometric ratios, nutrient concentrations of leaf litterfall and fresh leaves, and soil nutrient concentrations were determined by Spearman's correlation analysis. Analyses were conducted using Origin 8.0 software.

## 3 RESULTS

### 3.1 Nutrient concentrations in leaf litterfall and fresh leaves

C, N, and P concentrations in leaf samples from different tree species were shown in Table 2. C concentrations were not significantly different between fresh leaves and leaf litterfall. However, N and P concentrations in fresh leaves were higher than that those in leaf litterfall samples of the corresponding species. There was no consistent variation in specific values of C, N, and P concentrations in leaf samples. The highest N concentrations were found in CA, whereas the highest P concentrations were found in CL both in litterfall and fresh leaves. In leaf litterfall samples, PM had the lowest N and P concentrations.

**Table 2:** C, N, and P concentrations in leaf litterfall samples and fresh leaves of dominant tree species investigated

Tree species	Sampling season	C concentration (g/kg)		N concentration (g/kg)		P concentration (g/kg)	
		Leaf litterfall	Fresh leaves	Leaf litterfall	Fresh leaves	Leaf litterfall	Fresh leaves
*C. lanceolata*	Growing	439.99 ± 36.36^a,b,c^	441.84 ± 13.03^a,b^	12.52 ± 1.68^c,d^	14.53 ± 1.37^b^	0.49 ± 0.11^a^	0.79 ± 0.20^a^
	Nongrowing	460.66 ± 51.93^a^	452.56 ± 15.85^a,b^	10.96 ± 2.39^e^	14.50 ± 2.76^b^	0.40 ± 0.09^b^	0.85 ± 0.04^a^
*P. massoniana*	Growing	434.16 ± 58.41^b,c^	414.30 ± 11.20^b,c^	8.38 ± 1.61^f^	15.53 ± 1.26^b^	0.27 ± 0.07^d^	0.54 ± 0.03^c^
	Nongrowing	460.27 ± 38.20^a^	440.94 ± 15.50^a,b^	8.13 ± 1.56^f^	15.95 ± 1.62^b^	0.20 ± 0.06^e^	0.61 ± 0.07^b,c^
*C. axillaris*	Growing	418.78 ± 20.80^c^	402.22 ± 19.67^c,d^	16.37 ± 1.90^a^	19.80 ± 4.74^a^	0.48 ± 0.10^a^	0.67 ± 0.11^b^
	Nongrowing	392.55 ± 33.59^d^		11.93 ± 2.69^d,e^		0.37 ± 0.08^b,c^	
*C. glauca*	Growing	380.06 ± 89.83^d^	398.92 ± 24.71^d^	14.16 ± 2.54^b^	14.82 ± 3.28^b^	0.32 ± 0.05^c,d^	0.54 ± 0.09^c,d^
	Nongrowing	425.33 ± 46.37^b^	437.96 ± 32.56^a,b^	13.41 ± 2.37^b,c^	17.36 ± 1.15^a,b^	0.26 ± 0.07^d^	0.44 ± 0.06^d^
*L. glaber*	Growing	420.69 ± 38.13^b^	397.21 ± 33.55^d^	11.42 ± 2.90^d,e^	15.27 ± 2.21^b^	0.25 ± 0.15^d,e^	0.49 ± 0.06^c,d^
	Nongrowing	447.05 ± 38.17^a,b^	458.56 ± 34.48^a^	14.13 ± 2.17^b^	17.09 ± 1.39^a,b^	0.25 ± 0.08^d^	0.41 ± 0.04^d^

In each column, values from the same sample type with different letters indicate significant differences at *p *<* *.05.

Seasonal changes in nutrient concentrations of fresh leaves were not apparent when considering the same tree species, while the seasonal variation pattern in leaf litterfall was that C concentrations increased with decreases in N and P concentrations in the nongrowing season. That is, the quality of litterfall in the growing season was better than that in the nongrowing season.

### 3.2 Stoichiometric ratios in leaf litterfall and fresh leaves

**Figure 2:**
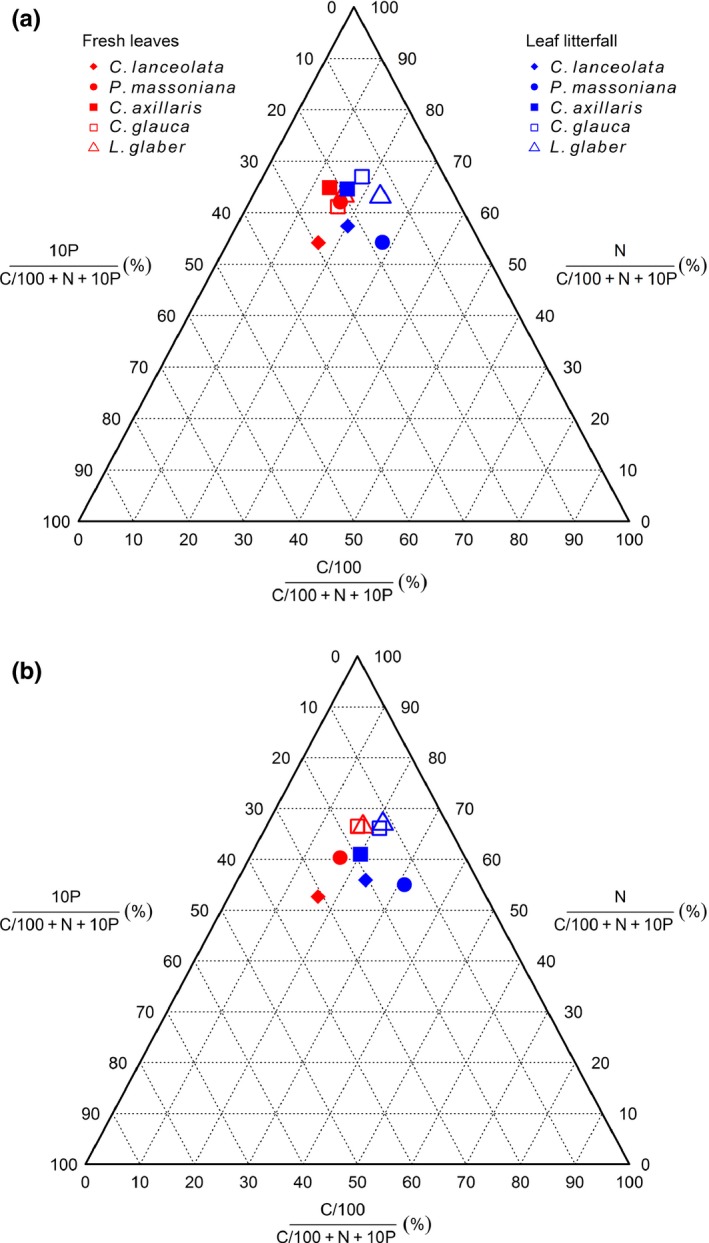
Ternary diagrams of C, N, and P in leaf litterfall and fresh leaves of dominant tree species investigated in the growing season (a) and nongrowing season (b). Data on fresh leaves of CA in the nongrowing season were omitted because there were no fresh leaves from deciduous species in this season. For visual reasons, the C concentrations were divided by a factor of 100, and the P concentration was multiplied by a factor of 10

The variation in the stoichiometric ratios C:N:P between leaf litterfall and fresh leaves was shown in a ternary diagram (Figure 2). For the five tree species investigated, relative C and P concentrations showed similar variation patterns in the growing and nongrowing seasons. To be specific, compared to leaf litterfall, the relevant symbols in fresh leaves moved to the left along the C axis and to the bottom left along the P axis, reflecting lower relative C concentrations accompanied by higher relative P concentrations in fresh leaves than in the corresponding leaf litterfall. The differences in the relative N concentrations between leaf litterfall and fresh leaves were smaller and did not have a consistent pattern. In all of the leaf samples, the relative N concentrations were in the interval between 50 and 70 along the N axis. In the growing season, the relative N concentration in fresh leaves of PM was higher than the value in litterfall, whereas the concentrations in fresh leaves of CL and CG were lower than those in leaf litterfall. In the nongrowing season, the relative N concentrations showed similar change patterns to the growing season, except for there being no obvious change in CG.

**Figure 3:**
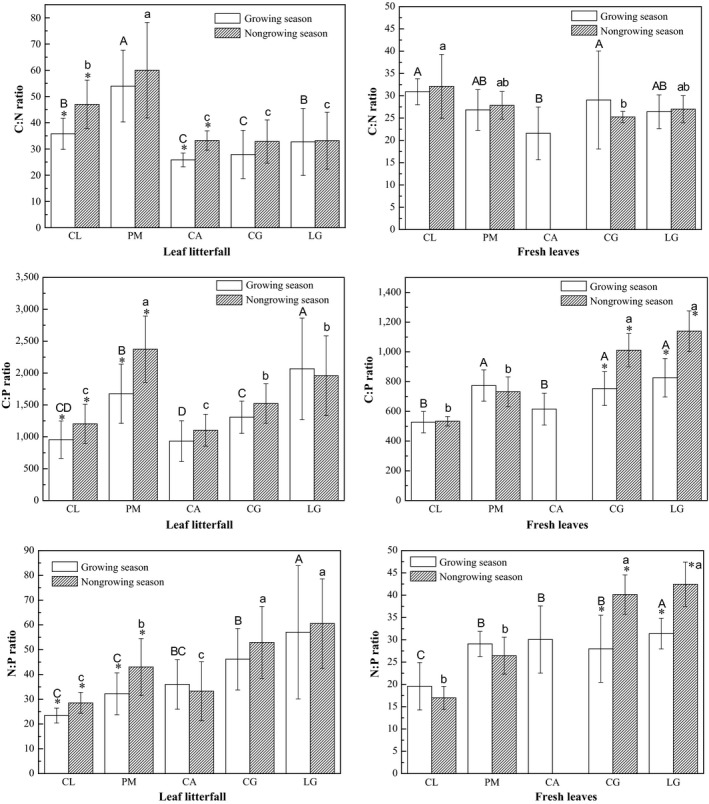
The seasonal variation in carbon: nitrogen (C:N), C:phosphorus (P), and N:P ratios in leaf litterfall and fresh leaves. Different upper and lower case letters indicate significant differences at *p *<* *.05 among tree species in growing and nongrowing seasons, respectively. Bars with * indicate significant differences at *p *<* *.05 between growing and nongrowing seasons for the same tree species. Data on fresh leaves of CA in the nongrowing season were omitted because there were no fresh leaves from deciduous species in this season. CL, PM, CA, CG, and LG denote *Cunninghamia lanceolata*,* Pinus massoniana*,* Choerospondias axillaris*,* Cyclobalanopsis glauca*, and *Lithocarpus glaber*, respectively

There was no consistent variation in C:N and C:P ratios among different species during successional development (Figure 3). However, similar variation tendencies of N:P ratios were obtained for fresh leaf and litterfall samples along successional stages, that was the ratios in LG from growing season, and those in CG and LG from nongrowing season were significantly higher than PM and CA. Seasonal variation in C, N, and P stoichiometric ratios in leaf samples was also shown in Figure 3. The C:N ratios of leaf samples were relatively stable except in the litterfall of CL and CA. Similar variation patterns were observed in the C:P and N:P ratios, and the variations differed from species to species. In particular, for CL and PM, the C:P and N:P ratios were relatively stable in fresh leaves, whereas they varied with the season in leaf litterfall. Conversely, for CG and LG, the C:P and N:P ratios were stable in leaf litterfall, whereas they varied with the season in fresh leaves. Overall, the stoichiometry ratios in leaf samples from the nongrowing season were significantly higher (*p* < .05) than the corresponding ratios from the growing season, which indicates that in the nongrowing season, C concentrations in both litterfall and fresh leaves were higher than N (C:N) and P (C:P) concentrations, and N concentrations were higher than P concentrations (N:P).

### 3.3 Nutrient resorption characteristics of different tree species

**Figure 4:**
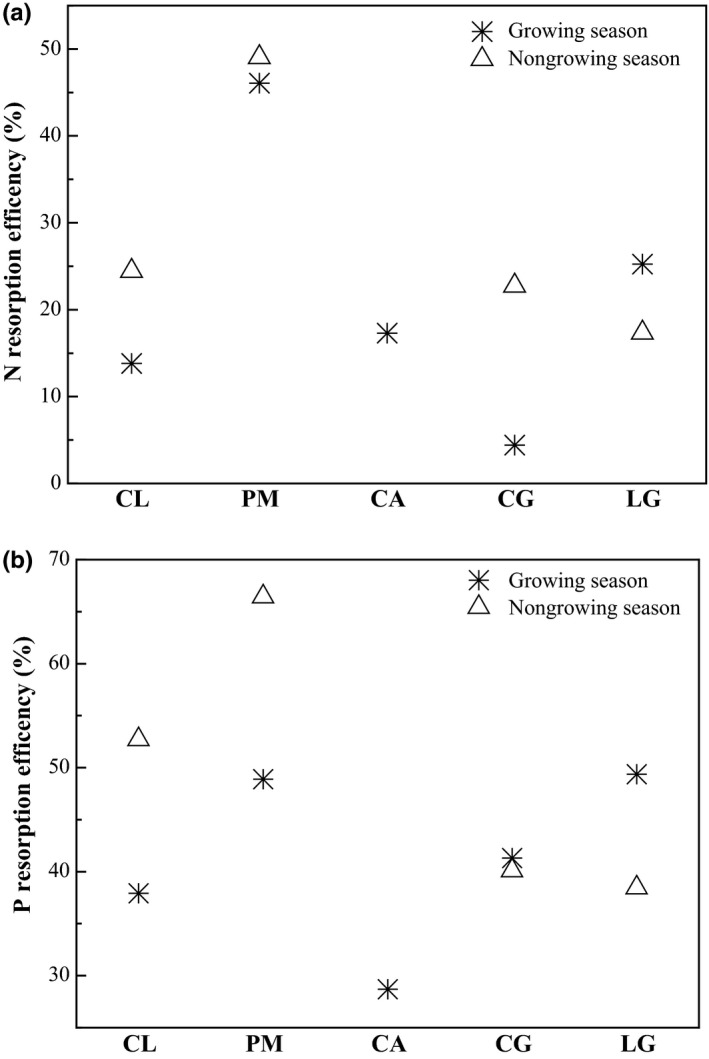
Nutrient resorption efficiency (RE) of N (a) and P (b) of different tree species investigated. Data on fresh leaves of CA in the nongrowing season were omitted because there were no fresh leaves from deciduous species in this season. CL, PM, CA, CG, and LG denote *Cunninghamia lanceolata*,* Pinus massoniana*,* Choerospondias axillaris*,* Cyclobalanopsis glauca*, and *Lithocarpus glaber*, respectively

In general, more than 60% of nutrients can be resorbed from litterfall and N REs in different tree species may vary within wide limits (Vergutz, Manzoni, Porporato, Novais, & Jackson, 2012). In this study, NREs ranged from 5% to 50%, while the range of variation in PREs was narrower (30%–70%) (Figure 4). The REs of P (P_RE_) were higher for the five tree species than the corresponding REs of N (N_RE_). However, compared to the other two species in which the values of P_RE_ were close to the N_RE_, P_RE_ for CL, LG, and CG were much more higher than the corresponding N_RE_. The N_RE_ and P_RE_ of PM were highest, and additionally, the highest N_RE_ among the other three tree species were only half (25.23%, LG in growing season) that of the highest N_RE_ in PM (49.04%, in nongrowing season). The lowest N_RE_ and P_RE_ were observed in the growing season in CG and CA, respectively.

There were no consistent patterns of seasonal changes in gross weight of leaf litterfall, and the deciduous tree species CA had the highest gross weight both in the growing and nongrowing seasons (Table 3). Therefore, the return amounts of N and P in CA were the highest among the five species investigated. In the growing season, the lowest return amounts of N and P were found in CG, and in the nongrowing season, the lowest values were found in LG, corresponding to the lowest gross weights of leaf litterfall in the growing and nongrowing season, respectively.

**Table 3:** Total dry weight of leaf litterfall and N and P return amounts via litterfall of dominant tree species investigated

Tree species	Sampling season	Total dry weight of leaf litterfall (g/m^2^)	N return amount (kg/ha)	P return amount (kg/ha)
*C. lanceolata*	Growing	75.46 ± 8.82	9.45	0.37
	Nongrowing	178.36 ± 13.56	19.54	0.72
*P. massoniana*	Growing	190.80 ± 21.90	15.98	0.52
	Nongrowing	122.23 ± 14.33	9.93	0.25
*C. axillaris*	Growing	203.81 ± 45.11	33.36	0.97
	Nongrowing	220.95 ± 55.21	26.36	0.82
*C. glauca*	Growing	42.73 ± 7.54	6.05	0.13
	Nongrowing	82.10 ± 25.33	11.01	0.21
*L. glaber*	Growing	86.79 ± 9.47	9.91	0.21
	Nongrowing	35.25 ± 4.69	4.98	0.08

### 3.4 Correlations among N and P resorption efficiencies, stoichiometric N:P ratios, and nutrient concentrations of leaf and soil samples

In all five species, N_RE_ and P_RE_ showed a significant and positive relationship with each other, and both were extremely negatively correlated with N concentrations in leaf litterfall (Table 4). Nutrients REs were weakly correlated with nutrient concentrations in soil.

**Table 4:** Spearman's correlation coefficients among N, P resorption efficiencies (subscript RE), stoichiometric N:P ratios of leaf litterfall (subscript L) and fresh leaves (subscript F), nutrient concentrations of leaf litterfall (subscript L) and fresh leaves (subscript F), and soil nutrient concentrations (subscript S) at *p* < .05 (*) or at *p *<* *.01 (**)

	N_RE_	P_RE_	N:P_L_	N:P_F_	C_L_	C_F_	N_L_	N_F_	P_L_	P_F_	C_S_	N_S_
P_RE_	0.75*											
N:P_L_	0.05	0.02										
N:P_F_	−0.02	−0.37	0.83**									
C_L_	0.48	0.43	−0.21	−0.38								
C_F_	0.03	−0.03	−0.20	−0.18	0.85**							
N_L_	−0.85**	−0.80**	0.32	0.43	−0.55	−0.20						
N_F_	0.08	−0.40	0.48	0.73*	−0.28	−0.12	0.37					
P_L_	−0.64	−0.55	−0.74*	−0.47	−0.20	0.02	0.36	−0.33				
P_F_	−0.08	0.09	−0.90**	−0.90**	0.28	0.15	−0.22	−0.50	0.66*			
C_S_	−0.30	−0.25	0.25	0.28	−0.63*	−0.79**	0.37	−0.03	0.03	−0.28		
N_S_	−0.37	−0.48	0.23	0.42	−0.84**	−0.81**	0.50	0.33	0.16	−0.24	0.85**	
P_S_	0.18	−0.04	−0.56	−0.42	−0.27	−0.29	−0.15	0.08	0.41	0.59	0.07	0.37

Stoichiometric N:P ratios in leaf litterfall and in fresh leaves were extremely positively correlated with each other, and both were extremely significantly negatively correlated with P concentrations in fresh leaves. In addition, N:P ratios in litterfall were significantly negatively correlated with P concentrations in litterfall and in fresh leaves, and the ratios were significantly positively correlated with N concentrations in fresh leaves. As with nutrient REs, the stoichiometric N:P ratios were weakly correlated with nutrient concentrations in soil.

C concentrations in leaf litterfall and in fresh leaves were positively correlated with each other, and both were negatively correlated with C and N concentrations in soil. P concentrations in leaf litterfall and in fresh leaves were positively correlated with each other. C concentrations in soil were extremely positively correlated with soil N concentrations. Apart from the above‐mentioned cases, the correlations between nutrient concentrations in leaves and in soil were weak.

## 4 DISCUSSION

### 4.1 Variations of nutrient concentrations and stoichiometric ratios in leaves

The results partially consistent with our hypothesis. That is, the variation of leaf C:N and C:P ratios was not consistent, whereas leaf N:P ratios increase from early‐ to late‐successional species owing to the increase in P limitation during forest successional development (Huang et al., 2013; Zhang et al., 2015). In fresh leaves, there was a pattern of higher leaf N:P ratios in the late‐successional tree species, indicating that the late‐successional species were more limited by P than by N. Moreover, in litterfall, the N:P ratios in LG and CG were much more higher than those in the other two species both in growing and nongrowing seasons, which implies that the late‐successional species were more likely to recycle P than N before litterfall. Corresponding to above‐mentioned results, soil P concentration at 0–30 cm depths in late‐stage forest was significantly lower than the early‐ and midsuccessional forests, whereas the soil N concentrations were not significantly changed during successional stages.

In leaf litterfall, the seasonal variation of N, P concentrations in deciduous species CA was more pronounced than that of the other four evergreens, and the nutrient concentrations in growing season were significantly higher than those in nongrowing season. This can be explained in this way: In the growing season, trees put forth more new leaves and have higher turnover rates; therefore, there is not enough energy to resorb nutrients at high level (Wang & Moore, 2014; Wright & Cannon, 2001). Compared to deciduous species, such as CA, evergreen species put forth new leaves gradually over the year. There were no significant seasonal variations of nutrient concentrations in fresh leaves, and the correlations between N and P concentrations in fresh leaves and in soils were weak, suggesting a strong homeostasis of fresh leaf nutrients in all the dominant tree species investigated (Wang & Moore, 2014).

Seasonal variations in stoichiometric ratios were much different from those in individual nutrient concentrations. The stoichiometric ratios of litterfall in CA were relatively stable except for C:N ratios, whereas the seasonal patterns of C:P and N:P ratios in the other four evergreens were varied depending on tree species. The two above‐mentioned ratios varied with seasons in leaf litterfall for CL and PM, whereas in fresh leaves for CG and LG. These patterns might be explained by the different nutrient use strategies of the different tree species (Yan et al., 2006).

### 4.2 Nutrient limitation in this region and resorption characteristics of different tree species

We found that all N:P ratios in fresh leaves were greater than 16 and the concentrations of P were lower than 1.0 mg/g, which indicating P limitation in this study area. This supports the general results obtained from southern China (Yan et al., 2006; Zeng et al., 2014; Zhang et al., 2015). Our results proved that the nutrient resorption characteristics were closely associated with nutrient limitation. In this P limitation study area, the P_RE_ was higher than the corresponding N_RE_. Meanwhile, all the five tree species investigated had high RP of P, as indicated by the results that N concentrations in litterfall were all higher than 7.0 mg/g, whereas the P concentrations were all lower than 0.5 mg/g (Killingbeck, 1996). Moreover, the same tree species PM in Tiantong National Forest Park had higher N_RE_ than the corresponding P_RE_, which was consistent with N limitation in the forest (Yan et al., 2006). Thus, it is surmisable that the nutrient limitation condition is a more important decision factor on the priority of nutrient resorption than tree species. However, N and P resorption characteristics (REs and RPs) were weakly correlated with nutrient concentrations in soil (Aerts, 1996; Aerts & Chapin, 2000; Norris & Reich, 2009). This may be explained by the relatively similar soil environment among four forests and the significant differences in nutrient resorption ability among five tree species.

### 4.3 Nutrient use strategies at different successional stages

Tree species PM not only to maximize percent reduction between fresh and senesced leaves (RE), but also to minimize terminal content in senesced leaves (RP), along with the results of seasonal variation in C:P and N:P stoichiometric ratios, PM adopted a “conservative consumption” nutrient use strategy in soil conditions of P limitation, which may increase endurance in an infertile environment (Aerts & Chapin, 2000; Escudero et al., 1992; Kobe et al., 2005; Wright & Cannon, 2001). It seems that PM, mainly through the regulation of P resorption from litterfall, maintained relatively stable C:P and N:P ratios in fresh leaves. Compared to PM, CG and LG took up nutrients via a “resource spending” strategy. Nutrient RE and RP in these two tree species were much lower than in PM, and the C:P and N:P ratios were stable in litterfall and varied with the season in fresh leaves. However, the attributes of CA did not fall between PM, and CG and LG, which may contribute to its deciduous leaf traits that lead to a higher new leaf turnover rate and more costly nutrient resorption in the growing season (Wang, Murphy, & Moore, 2014; Wright & Cannon, 2001). Therefore, considering dominant tree species in the three naturally restored secondary forests, distinguishing nutrient use strategies were adopted at different successional stages.

Based on our results, tree species at late‐successional stage tended to have the relatively open cycling of major nutrients, whereas at early‐successional stage cycled nutrients tightly, which is consistent with Yan's finding in Tiantong Forest Park (Yan et al., 2006) but contradictory to the Odum's hypothesis. One of the possible explanation for the contradiction was that the Odum's hypothesis and the expected trends were base on the observation in the holistic successional development of ecosystems from abandoned land during grassland and shrubland to forests (Eugene, 1969; Garnier et al., 2004; Vile et al., 2006). Compared to shrub or herbaceous plant, the forest successional stages in this study were relatively mature systems so that the variation tendency was different from Odum's observation and the nutrient cycling were all relatively tightening than pioneer stages. Another possible explanation was a new theory proposed by Friederike (Friederike et al., 2016) that not maturity of the ecosystem per se*,* but the P status of soils is the main driver of the tightness of P cycling. According to this hypothesis, tight P recycling is a crucial emergent property of forest ecosystems established at P poor sites. As in this study, the P cycling in all stages was more tight than corresponding N cycling under P limitation conditions.

## 5 CONCLUSIONS

From early‐ to late‐successional species, the N:P ratios became significantly higher both in fresh leaves and litterfall, which implied that the P limitation increases during forest successional development and the late‐successional species were more likely to recycle P than N, respectively. P_RE_ and P_RP_ were higher than the N resorption parameters in all the tree species studied owing to the P limitation in this area. Furthermore, dominant tree species in secondary forests along a succession gradient adopted different nutrient use strategies, and the tendency from the early‐ to late‐successional stages was from “conservative consumption” to “resource spending.” However, the attributes of the intermediate successional species did not fall between those of the early‐ and the two late‐successional species.

## CONFLICT OF INTEREST

None declared.

## AUTHOR CONTRIBUTIONS

There are five co‐authors contributed to this manuscript and the contributions of each co‐author were as follows: XF, WX, and YZ involved in idea and study design; XF, YZ, and XD with support of WX and CP involved in data collection and analysis; YZ, WX, and CP involved in manuscript writing. All authors have approved the manuscript and agree with submission to *Ecology and Evolution*.

## References

[ece33527-bib-0001] Aerts, R. (1996). Nutrient resorption from senescing leaves of perennials: Are there general patterns? Journal of Ecology, 84, 597–608. https://doi.org/10.2307/2261481

[ece33527-bib-0002] Aerts, R. , & Chapin, F. S. (2000). The mineral nutrition of wild plants revisited: A re‐evaluation of processes and patterns. Advances in Ecological Research, 30, 1–67. https://doi.org/10.1016/S0065-2504(08)60016-1

[ece33527-bib-0003] Chapin, F. (1980). The mineral nutrition of wild plants. Annual Review of Ecology and Systematics, 11, 233–260. https://doi.org/10.1146/annurev.es.11.110180.001313

[ece33527-bib-0004] Clevelan, C. , & Liptzin, D. (2007). C:N: P stoichiometry in soil: Is there a “Redfield ration” for the microbial biomass? Biogeochemistry, 85, 235–252. https://doi.org/10.1007/s10533-007-9132-0

[ece33527-bib-0005] Côté, B. , Fyles, J.W. , & Djalilvand, H. (2002). Increasing N and P resorption efficiency and proficiency in northern deciduous hardwoods with decreasing foliar N and P concentrations. Annals of Forest Science, 59, 275–281. https://doi.org/10.1051/forest:2002023

[ece33527-bib-0006] Davidson, E. , de Carvalho, C. , Figueria, A. , Ishida, F. , Ometto, J. , Nardoto, G. , … Martinelli, L. (2007). Recuperation of nitrogen cycling in Amazonian forests following agricultural abandonment. Nature, 447, 995–998. https://doi.org/10.1038/nature05900 17581583 10.1038/nature05900

[ece33527-bib-0007] Du, Y. , Pan, G. , Li, L. , Hu, Z. , & Wang, X. (2011). Leaf N/P ratio and nutrient reuse between dominant species and stands: Predicting phosphorus deficiencies in Karst ecosystems, southwestern China. Environ Earth Science, 64, 299–309. https://doi.org/10.1007/s12665-010-0847-1

[ece33527-bib-0008] Eckstein, R. L. , & Karlsson, P. S. (1997). Above‐ground growth and nutrient use by plants in a subarctic environment: Effects of habitat, life‐from and species. Oikos, 79, 311–324. https://doi.org/10.2307/3546015

[ece33527-bib-0009] Eckstein, R. L. , Karlsson, P. S. , & Weih, M. (1999). Leaf life span and nutrient resorption as determinants of plant nutrient conservation in temperate‐arctic regions. New Phytologist, 143, 177–189. https://doi.org/10.1046/j.1469-8137.1999.00429.x

[ece33527-bib-0010] Elser, J. , Sterner, R. , Gorokhova, E. , Fagan, W. , Markow, T. , Cotner, J. , … Weide, L. (2000). Biological stoichiometry from genes to ecosystems. Ecological letters, 3, 540–550. https://doi.org/10.1046/j.1461-0248.2000.00185.x

[ece33527-bib-0011] Escudero, A. , Arco, J. M. D. , Sanz, I. C. , & Ayala, J. (1992). Effects of leaf longevity and retranslocation efficiency on the retention time of nutrients. Oecologia, 90, 80–87. https://doi.org/10.1007/BF00317812 28312274 10.1007/BF00317812

[ece33527-bib-0013] Friederike, L. , Jurgen, B. , Emmanuel, F. , Eckhard, G. , Klaus, K. , Martin, K. , … Nicole, W. (2016). Phosphorus in forest ecosystems: New insights from an ecosystem nutrition perspective. Journal of Plant Nutrition and Soil Science, 179, 129–135. https://doi.org/10.1002/jpln.201500541

[ece33527-bib-0014] Garnier, E. , Cortez, J. , Billes, G. , Marie‐Laure, N. , Catherine, R. , Max, D. , … Jean‐Patrick, T. (2004). Plant functional markers capture ecosystem properties during secondary succession. Ecology, 85, 2630–2637. https://doi.org/10.1890/03-0799

[ece33527-bib-0015] Hagen‐Thorn, A. , Armolaitis, K. S. , Callesen, I. , & Stjernquist, I. (2004). Macronutrients in tree stems and foliage: A comparative study of six temperate forest species planted at the same sites. Annals of Forest Science, 61, 489–498. https://doi.org/10.1051/forest:2004043

[ece33527-bib-0016] Han, W. , Fang, J. , Guo, D. , & Zhang, Y. (2005). Leaf nitrogen and phosphorus stoichiometry across 753 terrestrial plant species in China. New Phytologist, 1682, 377–385. https://doi.org/10.1111/j.1469-8137.2005.01530.x 10.1111/j.1469-8137.2005.01530.x 16219077

[ece33527-bib-0017] He, J. , Fang, J. , Wang, Z. , Guo, D. , Flynn, D. , & Gerg, Z. (2006). Stoichiometry and large‐scale patterns of leaf carbon and nitrogen in the grassland biomes of China. Oecologia, 149, 115–122. https://doi.org/10.1007/s00442-006-0425-0 16639565 10.1007/s00442-006-0425-0

[ece33527-bib-0018] He, J. , Wang, L. , Flynn, D. F. B. , Wang, X. , Ma, W. , & Fang, J. (2008). Leaf nitrogen: Phosphorus stoichiometry across Chinese grassland biomes. Oecologia, 155, 301–310. https://doi.org/10.1007/s00442-007-0912-y 18278518 10.1007/s00442-007-0912-y

[ece33527-bib-0019] Huang, W. , Liu, J. , Wang, Y. , Zhou, G. , Han, T. , & Li, Y. (2013). Increasing phosphorus limitation along three successional forests in southern China. Plant and Soil, 364, 181–191. https://doi.org/10.1007/s11104-012-1355-8

[ece33527-bib-0020] Kazakou, E. , Garnier, E. , Roumet, C. , Collin, C. , & Laurent, G. (2007). Components of nutrient residence time and the leaf economics spectrum in species from Mediterranean old‐fields differing in successional status. Functional Ecology, 21, 235–245. https://doi.org/10.1111/fec.2007.21.issue-2

[ece33527-bib-0021] Killingbeck, K. (1996). Nutrients in senesced leaves: Keys to the search for potential resorption and resorption proficiency. Journal of Ecology, 77, 1716–1727. https://doi.org/10.2307/2265777

[ece33527-bib-0022] Killingbeck, K. , Hammenwinn, S. , Vecchio, P. , & Goguen, M. (2002). Nutrient resorption efficiency and proficiency in fronds and trophopods of a winter‐deciduous fern, *Dennstaedtia punctilobula* . International Journal of Plant Sciences, 163, 99–105. https://doi.org/10.1086/324181

[ece33527-bib-0023] Kobe, R. K. , Lepczyk, C. A. , & Iyer, M. (2005). Resorption efficiency decrease with increasing green leaf nutrients in a global data set. Ecology, 86, 2780–2792. https://doi.org/10.1890/04-1830

[ece33527-bib-0024] Koerselman, W. , & Meuleman, A. (1996). The vegetation N: P ratio: A new tool to detect the nature of nutrient limitation. Journal of Applied Ecology, 33, 1441–1450. https://doi.org/10.2307/2404783

[ece33527-bib-0025] McGroddy, M. E. , Daufresne, T. , & Hedin, L. O. (2004). Scaling of C:N:P stoichiometry in forests worldwide: implications of terrestrial redfield‐type ratios. Ecological Society of America, 89, 2390–2401. http://doi.org/10.1890/03-0351

[ece33527-bib-0026] Milla, R. , Palacio‐Blasco, S. , Maestro‐Martinez, M. , & Montserrat‐Marti, G. (2006). Phosphorus accretion in old leaves of a Mediterranean shrub growing at a phosphorus‐rich site. Plant and Soil, 280, 369–372. https://doi.org/10.1007/s11104-005-3529-0

[ece33527-bib-0027] Norris, M. , & Reich, P. (2009). Modest enhancement of nitrogen conservation via retranslocation in response to gradients in N supply and leaf N status. Plant and Soil, 316, 193–204. https://doi.org/10.1007/s11104-008-9770-6

[ece33527-bib-0012] Odum, EP. (1969). The strategy of ecosystem development. Science, 164, 262–270. 5776636 10.1126/science.164.3877.262

[ece33527-bib-0028] Pan, R. (2006). Plant physiology (pp. 50–70). Beijing: Chemical Industry Press.

[ece33527-bib-0029] Pugnaire, F. , & Chapin, F. (1993). Controls over nutrient resorption from leaves of evergreen Mediterranean species. Ecology, 74, 124–129. https://doi.org/10.2307/1939507

[ece33527-bib-0030] Regina, I. , Rico, M. , Rapp, M. , & Gallego, H. (1997). Seasonal variation in nutrient concentration in leaves and branches of *Quercus pyrenaica* . Journal of Vegetation Science, 8, 651–654. https://doi.org/10.2307/3237369

[ece33527-bib-0031] Reich, P. B. , & Oleksyn, J. (2004). Global patterns of plant leaf N and P in relation to temperature and latitude. Proceedings of the National Academy of the United States, 101, 11001–11006. https://doi.org/10.1073/pnas.0403588101 10.1073/pnas.0403588101 PMC503733 15213326

[ece33527-bib-0032] Reich, P. B. , Walters, M. B. , & Ellsworth, D. S. (1992). Leaf lifespan in relation to leaf, plant and stand characteristics among diverse ecosystem. Ecological Monographs, 62, 365–392. https://doi.org/10.2307/2937116

[ece33527-bib-0033] Robert, B. , Caritat, A. , Bertoni, G. , Vilar, L. , & Molinas, M. (1996). Nutrient content and seasonal fluctuations in the leaf component of cork‐oak (*Quercus suber L*.) litterfall. Plant Ecology, 122, 29–35. https://doi.org/10.1007/BF00052813

[ece33527-bib-0034] Salehi, A. , Ghorbanzadeh, N. , & Salehi, M. (2014). Soil nutrient status, nutrient return and retranslocation in poplar species and clones in northern Iran. iForest Biogeosciences and Forestry, 6, 336–341. https://doi.org/10.3832/ifor0976-006

[ece33527-bib-0035] Sterner, R. , & Elser, J. (2002). Ecological stoichiometry: The biology of elements from molecules to the biosphere (p. 439). Prinston, NJ: Princeton University Press.

[ece33527-bib-0036] Tessier, J. , & Raynal, D. (2003). Use of nitrogen to phosphorus ratios in plant tissue as an indicator of nutrient limitation and nitrogen saturation. Journal of Applied Ecology, 40, 523–534. https://doi.org/10.1046/j.1365-2664.2003.00820.x

[ece33527-bib-0037] Vergutz, L. , Manzoni, S. , Porporato, A. , Novais, R. F. , & Jackson, R. B. (2012). Global resorption efficiencies and concentrations of carbon and nutrients in leaves of terrestrial plants. Ecological Monographs, 82, 205–220. https://doi.org/10.1890/11-0416.1

[ece33527-bib-0038] Verhoeven, J. , Koerselman, W. , & Meuleman, A. (1996). Nitrogen‐ or phosphorus‐limited growth in herbaceous, wet vegetation: Relations with atmospheric inputs and management regimes. Trends in Ecology and Evolution, 11, 494–497. https://doi.org/10.1016/S0169-5347(96)10055-0 21237936 10.1016/s0169-5347(96)10055-0

[ece33527-bib-0039] Victor, A. , Dimitrios, A. , Alexandros, T. , Georgios, B. , & Georgios, S. (2001). Litterfall, litter accumulation and litter decomposition rates in four forest ecosystems in northern Greece. Forest Ecology and Management, 144, 113–127. https://doi.org/10.1016/s0378-1127(00)00365-0

[ece33527-bib-0040] Vile, D. , Shipley, B. , & Garnier, E. (2006). A structural equation model to integrate changes in functional strategies during old‐field succession. Ecology, 87, 504–517. https://doi.org/10.1890/05-0822 16637374 10.1890/05-0822

[ece33527-bib-0041] Wang, M. , & Moore, T. (2014). Carbon, nitrogen, phosphorus, and potassium stoichiometry in an ombrotrophic peatland reflects plant functional type. Ecosystems, 17, 673–684. https://doi.org/10.1007/s10021-014-9752-x

[ece33527-bib-0042] Wang, M. , Murphy, M. , & Moore, T. (2014). Nutrient resorption of two evergreen shrubs in response to long‐term fertilization in a bog. Oecologia, 174, 365–377. https://doi.org/10.1007/s00442-013-2784-7 24078082 10.1007/s00442-013-2784-7

[ece33527-bib-0043] Wardle, D. , Walker, L. , & Bardgett, R. (2004). Ecosystem properties and forest decline in contrasting long‐term chronosequences. Science, 305, 509–513. https://doi.org/10.1126/science.1098778 15205475 10.1126/science.1098778

[ece33527-bib-0044] Wright, I. J. , & Cannon, K. (2001). Relationships between leaf lifespan and structural defences in a low‐nutrient, sclerophyll flora. Functional Ecology, 15, 351–359. https://doi.org/10.1046/j.1365-2435.2001.00522.x

[ece33527-bib-0045] Xiang, W. , Chai, H. , Tian, D. , & Peng, C. (2009). Marginal effects of silvicultural treatments on soil nutrients following harvest in a Chinese fir plantation. Soil Science and Plant Nutrition, 55, 523–531. https://doi.org/10.1111/j.1747-0765.2009.00384.x

[ece33527-bib-0046] Yan, E. , Wang, X. , & Huang, J. (2006). Shifts in plant nutrient use strategies under secondary forest succession. Plant and Soil, 289, 187–197. https://doi.org/10.1007/s11104-006-9128-x

[ece33527-bib-0047] Yuan, Z. , Li, L. , Han, X. , Huang, J. , & Jiang, G. (2005). Nitrogen resorption from senescing leaves in 28 plant species in a semi‐arid region of northern China. Journal of Arid Environments, 63, 191–202. https://doi.org/10.1016/j.jaridenv.2005.01.023

[ece33527-bib-0048] Yuan, Z. , & Wan, S. (2005). Soil characteristics and nitrogen resorption in *Stipa krylovii* native to northern China. Plant and Soil, 273, 257–268. https://doi.org/10.1007/s11104-004-7941-7

[ece33527-bib-0049] Zeng, Y. , Xiang, W. , Deng, X. , Fang, X. , Liu, C. , & Peng, C. (2014). Soil N forms and gross transformation rates in Chinese subtropical forests dominated by different tree species. Plant and Soil, 384, 231–242. https://doi.org/10.1007/s11104-014-2206-6

[ece33527-bib-0050] Zhang, W. , Zhao, J. , Pan, F. , Li, D. , Chen, H. , & Wang, K. (2015). Changes in nitrogen and phosphorus limitation during secondary succession in a karst region in south west China. Plant and Soil, 391, 77–91. https://doi.org/10.1007/s11104-015-2406-8

